# Pelvic organ prolapse patients’ attitudes and preferences regarding their uterus: comparing German- and Russian-speaking women

**DOI:** 10.1007/s00192-019-03918-9

**Published:** 2019-04-26

**Authors:** Polina Lyatoshinsky, Christian Fünfgeld, Alexander Popov, Vitaly Bezhenar, Viktoria Krutova, Daniela Ulrich, Wolfgang Umek

**Affiliations:** 1 Department of Obstetrics and Gynecology, Medical University of Vienna, Vienna, Austria; 2 Department of Obstetrics and Gynecology, Danube Hospital, Vienna, Austria; 3 Tettnang Hospital, Tettnang, Baden-Württemberg Germany; 4 Moscow Regional Research Institute of Obstetrics and Gynecology, Moscow, Russia; 5 Pavlov State Medical University, Saint Petersburg, Russia; 6 Kuban State Medical University, Krasnodar, Russia; 7 Department of Obstetrics and Gynecology, Medical University of Graz, Graz, Austria; 8 Karl-Landsteiner-Institute of Specialized Obstetrics and Gynecology, Vienna, Austria

**Keywords:** Pelvic organ prolapse, Uterus-sparing prolapse surgery, Uterus score, Attitude, Preference

## Abstract

**Introduction and hypothesis:**

The aim of this study was to compare preferences of patients with pelvic organ prolapse (POP) regarding their uterus between German- and Russian-speaking areas.

**Methods:**

Six urogynecologic tertiary referral centers participated in this prospective study: three centers from German-speaking countries and three from different regions of Russia. To assess the uterus-related preferences as well as the attitude toward hysterectomy versus uterus-sparing prolapse surgery, we developed a structured questionnaire that included 5-point Likert scales related to benefit of uterus (BOU) and benefit of not having uterus (BNU). Each scale consisted of 12 items (range of possible scores: 12–60). Finally, patients were asked if they preferred uterus removal or preservation when undergoing prolapse surgery.

**Results:**

One hundred and seventy-eight German-speaking and 206 Russian-speaking patients were included in the study. There was no significant difference in patients’ preference before undergoing POP surgery regarding uterus preservation versus hysterectomy between German- and Russian-speaking patients: 40% of German-speaking and 54% of Russian-speaking patients preferred to retain their uterus before undergoing POP surgery.

Comparison of BOU mean scores showed a significant difference between groups: 20.6 ± 6.7 for German-speaking compared with 32.5 ± 9.1 for Russian-speaking patients (*p* < 0.01). The Russian-speaking group had significantly higher mean scores on domains sexuality, body image, and partnership of the BOU scale (2.6 ± 1.0 vs. 1.8 ± 0.9 for sexuality; 2.4 ± 1.1 vs. 1.5 ± 0.7 for body image, and 2.6 ± 0.9 vs. 1.6 ± 0.7 for partnership domains; *p* < 0.05).

**Conclusions:**

Although a large proportion of German- and Russian-speaking patients prefers uterus preservation when undergoing prolapse surgery, the uterus was more important for sexuality, partnership, and body image in Russian-speaking patients.

**Electronic supplementary material:**

The online version of this article (10.1007/s00192-019-03918-9) contains supplementary material, which is available to authorized users.

## Introduction

Although many women undergo hysterectomy for pelvic organ prolapse (POP), little is known about women’s preferences for either uterine preservation or hysterectomy. Studies from the USA showed that many women with symptomatic POP who presented for urogynecological evaluation preferred uterine preservation [1–3]. A study from The Netherlands showed that patients had a preference for uterine preservation when outcomes for preservation or hysterectomy were expected to be equal [4]. Other authors reported that women with prolapse had relatively neutral attitudes on whether the uterus was beneficial for their sexuality or femininity [5]. Previous studies have also evaluated the impact of uterine preservation and hysterectomy on sexual function, body image, and relationships [6–9]. Planning prolapse surgery in multicultural social environments is a challenge for both surgeons and patients. The aim of this study was to compare women’s preferences and attitudes toward their uterus between German- and Russian-speaking patients scheduled for POP surgery.

## Methods

Six academic tertiary referral prolapse centers participated in this prospective study. Three study centers were located in German-speaking countries: Medical University of Vienna and Graz in Austria, and Tettnang Hospital in Germany; three study centers were located in different regions of Russia: Moscow Regional Research Institute of Obstetrics and Gynecology, Pavlov State Medical University of Saint Petersburg, and Kuban State Medical University in Krasnodar.

All eligible patients with POP were asked to participate and were handed the questionnaires after consenting to participate. All patients had the opportunity to undergo prolapse surgery with or without uterus removal and were given detailed information about both procedures. For the purpose of the study, we assumed that the functional surgical outcome after uterine preservation or hysterectomy would be equal. Patients after hysterectomy, patients with concomitant diseases of the uterus that required surgical treatment, and patients without adequate language skills were excluded from the study. The ethics committees of all six study centers approved the study.

### Questionnaire

The study questionnaire included information on demography, level of education, previous experience with POP, and symptom severity. A 4-point scoring system was used to assess symptom severity (Pelvic Floor Questionnaire) [10]. Patients were also asked whether they had been seeking information about POP through printed sources, the Internet, broadcast media, family, friends, or healthcare providers. The Knowledge About Pelvic Organ Prolapse Questionnaire, validated for German- and Russian-speaking patients, was used to assess level of knowledge about POP (score 0–16) [11]. Sexually active patients were asked to complete the Female Sexual Function Index (FSFI) questionnaire written in their respective language [12, 13].

To assess uterus-related preferences and attitudes toward hysterectomy versus uterus-preserving prolapse surgery, we developed two structured 5-point-Likert scales: benefit of uterus (BOU) and benefit of not having uterus (BNU), each consisting of 12 items (Supplementary appendix). These scales were developed with the assistance of K. Leithner-Dzubias, head of the psychosomatic outpatient clinic at the Medical University of Vienna, based on existing surveys about women’s perceptions about hysterectomy and body image [7, 14–19]. Five items were taken from the scoring system used by Good et al. [5], five from the survey composed by Frick et al. [1], and three from the study by Kuppermann et al. [19]. Patients were asked if the uterus was important for their sexual function and emotional state or if removing the uterus would worsen their relationship or their body image. A higher median score on the BOU scale indicated greater benefit for uterine preservation. The higher median score on the BNU scale indicated greater benefit of removing the uterus. Finally, patients were asked if they would prefer to preserve or remove their uterus when undergoing prolapse surgery. The 5-point Control Preference Scale (CPS) validated by Sung et al. [20] was used to assess patients’ preferences regarding their role in making a decision about the treatment of POP.

We categorized patients as active in their treatment decision-making process if they responded they preferred to make the final decision or the final decision after seriously considering the doctor’s opinion. Those who responded yes were categorized as taking a collaborative role. Those responding that they preferred the doctor make the decision or that they would like the physician to make the decision after considering their opinion were categorized as taking a passive role [1, 20]. Logistic regression analysis was applied to identify the contribution of demographic variables, level of knowledge about POP, and scores of uterus-related benefit scales.

## Results

A total of 203 German- and 212 Russian-speaking women were eligible to participate in the study between September 2016 and May 2018. Seventeen and six patients from the respective groups declined enrollment. Eight German-speaking women were excluded after seeing the physician: three for incorrect hysterectomy status and five because of incorrect referral diagnosis of POP. One hundred and seventy-eight German-speaking and 206 Russian-speaking women with POP were included in the final analysis.

Patient demographics are presented in Table 1. Russian-speaking patients were younger and more likely to have a higher level of education. Parity and subjective severity of POP symptoms were significantly higher in the German-speaking group. Twenty-one percent of German- and 10% of Russia-speaking patients were diagnosed with POP relapse after previous surgery (*p* < 0.05).

**Table 1:** Demographic characteristics and pelvic organ prolapse (POP) history of study participants

	German-speaking patients (*n* = 178)	Russian-speaking patients (*n* = 206)
Age (years), mean ± SD	60.9 ± 9.5	55.6 ± 9.3*
Marital status, % (*n*)		
Married	62 (112)	68 (139)
Single	6 (10)	5 (11)
Divorced	16 (28)	12 (24)
Widowed	16 (28)	15 (32)
Highest level of education, % (*n*)		
Secondary school	58 (104)	5 (11)*
Undergraduate	26 (46)	40 (83)*
Graduate school	16 (28)	55 (112)*
Postmenopausal status, % (*n*)	81 (144)	63 (130)
Patients’ history of cancer, % (*n*)	13 (23)	7 (14)
Family history of cancer, % (*n*)	28 (50)	24 (49)
Parity, mean ± SD	2.4 ± 0.9	1.8 ± 0.5*
Sexually active patients, % (*n*)	47 (83)	59 (121)
POP Symptom Severity Score, mean ± SD	2.4 ± 0.6	2.1 ± 0.6 *
Previous POP treatment, % (*n*)		
Conservative	29 (52)	14 (28) *
Surgery	12 (21)	5 (10) *
Prolapse Knowledge score (0–16), mean ± SD	11.4 ± 2.9	10.1 ± 2.3*
Patients’ sources of information about POP, % (*n*)		
Gynecologist	82 (146)	70 (145)
Friends and family	42 (74)	15 (31)*
Internet	32 (56)	20 (41)
General practitioner	19 (33)	15 (31)
Newspapers and magazines	27 (48)	5 (10)*
Radio and television	6 (11)	5 (11)

*SD* standard deviation

*p < 0.05

German-speaking patients were more informed about POP than Russian-speaking patients, with Prolapse Knowledge scores of 11.4 and 10.1, respectively (*p* < 0.05), and were more likely to use printed media sources and share information about POP with family and friends. The proportion of sexually active patients was not significantly different between groups (47% the German and 59% in the Russian group; *p* > 0.05). Assessment of sexual function using the FSFI showed a significantly lower total median score and lower scores of domains orgasm, satisfaction, and pain in the Russian- versus the the German group (Table 2).

**Table 2:** Median Female Sexual Function Index (FSFI) scores in sexually active participants

Domains (range)	German speaking (*n* = 83)	Russian speaking (*n* = 121)
Desire (1.2–6.0)	3.1 ± 0.9	3.3 ± 0.9
Arousal (0–6.0)	3.6 ± 1.1	3.5 ± 1.1
Lubrication (0–6.0)	4.2 ± 1.4	4.0 ± 1.4
Orgasm (0–6.0)	4.0 ± 1.3	3.3 ± 1.4*
Satisfaction (0.8–6.0)	4.2 ± 1.2	3.4 ± 0.9*
Pain (0–6.0)	4.5 ± 1.4	4.0 ± 1.5*
All items (2.0–36.0)	23.6 ± 6.2	21.4 ± 5.2*

*p < 0.05

### Analysis of uterus-related preferences

**Fig. 1:**
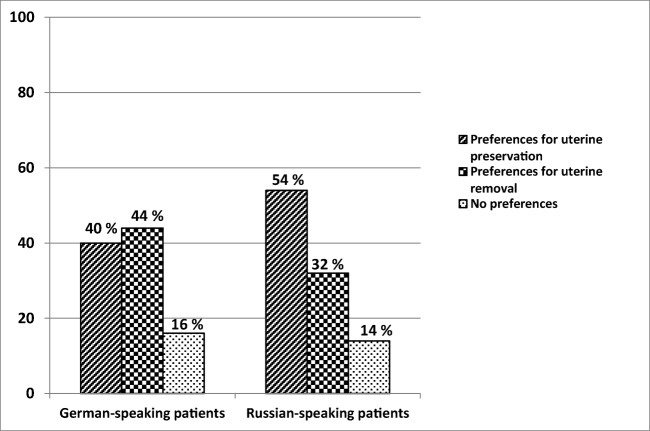
Patients’ preferences regarding uterus preservation at the time of prolapse surgery (*p* > 0.05)

Assuming the equal functional outcome of uterus-preserving surgery and hysterectomy, there was no significant difference in patients’ preferences between groups: 40% of German- and 54% of Russian-speaking patients preferred to retain their uterus; 44 and 32%, respectively, preferred hysterectomy (*p* > 0.05) (Fig. 1).

The proportion of women who stated that the uterus was important for their sexuality, body image, or partnership was lower in the German-speaking group (Table 3). Russian-speaking women were mostly concerned about feeling older and being sad about losing their fertility and body image when undergoing hysterectomy.

**Table 3:** Examples of uterus-related statements as part of Benefit-of-Uterus scale

Uterus-related statements	German speaking (*n* = 178)			Russian speaking (*n* = 206)		
	Strongly agree/agree	Neither agree/ disagree	Disagree/ strongly disagree	Strongly agree/ agree	Neither agree/ disagree	Disagree/ strongly disagree
The uterus is important for my:						
Sexuality	8 (14)	17 (31)	75 (133)	24 (50)*	32 (65)*	44 (91)*
Body image	14 (25)	16 (28)	70 (125)	28 (58)*	25 (52)	47 (96)*
Partnership	8 (15)	10 (18)	82 (145)	22 (46)*	40 (82)*	38 (78)*
Without uterus I would:						
Feel older	8 (15)	7 (12)	85 (151)	29 (59)*	20 (42)*	51 (105)*
Be sad about losing my fertility	9 (16)	7 (12)	84 (150)	29 (59)*	26 (54)*	45 (93)*

*p < 0.05, n% (*n*)

**Fig. 2:**
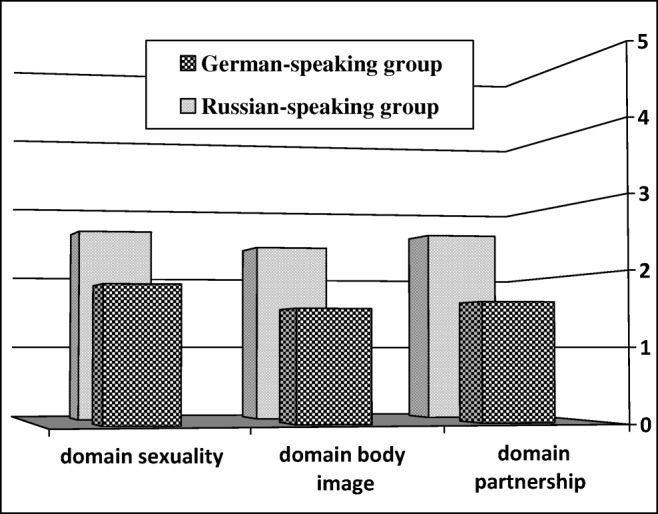
Mean scores of partnership, body image, and sexuality domains in the Benefit of Uterus (BOU) 5-point Likert scale between German- and Russian-speaking groups (*p* < 0.05).

With a mean BOU score of 32.5 ± 9.1, Russian-speaking patients saw a greater benefit for uterine preservation than German-speaking patients [20.6 ± 6.7, (*p* < 0.01)]. The Russian-speaking group had significantly higher mean scores on domains sexuality, body image, and partnership of the BOU scale [2.6 ± 1.0 vs. 1.8 ± 0.9 for sexuality; 2.4 ± 1.1 vs. 1.5 ± 0.7 for body image, 2.6 ± 0.9 vs. 1.6 ± 0.7 for partnership domains (*p* < 0.05)](Fig. 2).

Mean BNU scale scores did not differ between the German- and Russian-speaking women (29.6 ± 6.9 vs. 31.0 ± 6.5; *p* > 0.05). A psychometric analysis of the BOU and BONU scales showed an acceptable reliability in both groups. Cronbach’s alpha coefficient was 0.904 for the German the BOU scale and 0.916 for the Russian. Cronbach’s alpha coefficients for the BNU scale were 0.769 and 0.876, respectively.

### Analysis of the Control Preferences Scale (CPS)

Forty-one percent of German-speaking patients with POP preferred an active and 42% to a collaborative role in decision making. Russian-speaking patients mostly preferred the doctor to make a treatment decision (55% passive patients) (Table 4).

**Table 4:** The role of the patients in decision making regarding their treatment of pelvic organ prolapse (POP): Control Preference Scale (CPS)

	German-speaking group *n* = 178	Russian-speaking group *n* = 206
Active, % (*n*)	41 (73)	19 (38) *
Collaborative, % (*n*)	42 (75)	26 (54) *
Passive, % (*n*)	17 (30)	55 (114) *

*p < 0.05

### Predictive factors for patients’ decision regarding their uterus at the time of POP surgery

Logistic regression analysis demonstrated that significant predictors for a patient’s decision to preserve her uterus were higher BOU scores, higher level of education, higher POP knowledge scale scores, and use of the Internet. Predictors for decision to remove the uterus were being older, higher BNU scores, and history of cancer. Predictable values for all variables are presented in Table 5.

**Table 5:** Predictors for patients’ decision to preserve uterus at the time of the pelvic organ prolapse (POP) surgery

Predictors	Odds ratio	95% confidence interval
Age^a^	0.97	0.95–0.99
Patients’ level of knowledge about POP^a^	1.15	1.06–1.25
BOU score^a^	1.12	1.07–1.3
BNU score^a^	0.97	0.95–0.99
Education level^a^	1.8	1.2–2.9
Patients’ sources of information about POP		
Gynecologist	1.1	0.7–1.2
Friends and family	1.1	0.7–1.6
Internet^a^	1.7	1.1–2.7
General practitioner	0.9	0.6–1.6
Newspapers and magazines	1.3	0.8–2.3
Radio and television	1.4	0.7–3.1
Patients’ history of cancer^a^	0.2	0.1–0.5
Parity	1.1	0.8–1.3
POP symptoms severity	0.9	0.7–1.3
Previous treatment of POP	1.4	0.8–2.2
Control Preference Scale	1.1	0.8–2.0
Female Sexual Function Index	1.0	0.99–1.03
German- or Russian-speaking geographical area	0.5	0.2–1.2

Odds ratios and 95% confidence intervals were extracted from the logistic regression analysis

*BOU* benefit of uterus, *BNU* benefit of not having uterus

^a^If the confidence interval does not contain the relative risk of 1.0, the factor is significantly predictive for the decision making

## Discussion

In this study, we compared attitudes and preferences toward the uterus between German- and Russian-speaking women scheduled for prolapse surgery, assuming that outcomes after uterus-preserving surgery and hysterectomy were equal. Six study centers were located in Austria, Germany, and different regions of Russia. We developed BOU and BNU scales in German and Russian to assess patients’ perception about their uterus when undergoing prolapse surgery: preferences did not differ. A large proportion of women in both groups preferred to maintain their uterus (40% of German- and 54% of Russian-speaking patients).

Two studies from the USA demonstrated similar patient preferences. In the study by Korbly, 36% of women preferred uterine preservation, assuming surgical outcomes were equal [2]. In the study by Frick, 60% of women would have declined hysterectomy if presented with an equally efficacious surgical option [1]. In the Dutch population, 43% of women with prolapse expressed preferences for uterus preservation [4]. Conversely, the lower proportion (31%) who chose to preserve their uterus was reported in the Hispanic group with lower income in a study by Wong et al. [3].

In our study, Russian-speaking women demonstrated higher mean scores than German-speaking patients on the BOU scale and were more concerned about their sexuality, body image, partnership, feeling older, and losing their fertility. In a North American study by Good, patients showed neutral attitudes toward their uterus and, contrary to our results, most prolapse patients did not express importance of their uterus for body image and sexuality [5].

FSFI scores in both groups in our study were comparable with the scores presented by Nazapour, who investigated sexual function among postmenopausal women [21]. The different levels of sexual satisfaction between Russian- and German-speaking groups may be explained by the cultural differences in sexual relationships or social taboos around sexuality. We could not confirm that patients taking a more active part in the decision-making process were more likely to decline or agree to hysterectomy, as was shown by Frick et al. [1]. Neither FSFI scores nor prolapse severity was predictable for the decision regarding the uterus in our study.

Our logistic regression analysis revealed that significant predictors for a patient’s decision to preserve the uterus were younger age, higher BPU scores, higher level of education, and higher level of knowledge about prolapse. A predictable value of higher education for uterine preservation was also shown by Korbly et al. [2]. A limited educational level of Hispanic women who mostly preferred to remove their uterus was noticed in the study by Wong et al. [3], but the language spoken in a specific geographical area was not predictable.

The strength of our study was that we developed two reliable 12–item uterus-related preference scales in German and Russian that could help clinicians include patients’ perception about their uterus in the planning of prolapse surgery and make a shared treatment decision. A similar predictable scoring system was also demonstrated in a study by Kuppermann [19].

Our study has also some limitations. Demographic heterogeneity of study groups should be considered: Russian-speaking patients were younger and more likely to have a higher level of education than German-speaking patients. Selection bias cannot be excluded: Although all participating centers were tertiary referral centers, practices, selection criteria for surgery, and management of POP patients might have differed between centers. We did not collect data about ethnicity, origin, or religious affiliation of participants, which should be taken into concern in further research.

In conclusion, although a large proportion of German- and Russian-speaking patients preferred to maintain their uterus when undergoing POP surgery, the uterus was more important for sexuality, partnership, and body image in Russian-speaking patients. Generally, women undergoing prolapse surgery in Russia are younger and have a higher level of education than women in the German-speaking area, which could explain some bias in this study.

## Electronic supplementary material

ESM 1 (DOCX 117 kb)
