# Supplementary material for: Pelvic organ prolapse patients’ attitudes and preferences regarding their uterus: comparing German- and Russian-speaking women
Source: Int Urogynecol J. 2019 Apr 26;30(12):2077–83. doi: 10.1007/s00192-019-03918-9 (PMC6861192; doi:10.1007/s00192-019-03918-9)
Supplement: Supplementary file 1 — (DOCX 117 kb) [file 192_2019_3918_MOESM1_ESM.docx]

**Appendix.**

Likert items of “Benefit-of-uterus” and “Benefit-of not having-uterus” scales (translation from the German and Russian original versions). Five ordered response levels were used for each item (1-strongly disagree, 2-disagree, 3-neither agree nor disagree, 4-agree, 5-strongly agree).

| Items | “Benefit-of-uterus” scale |
| --- | --- |
| 1 | Having a uterus is important for my sense of self and my emotional state |
| 2 | A uterine removal would make me less feminine |
| 3 | I would feel my body was less whole without a uterus |
| 4 | The uterus is important for my sexuality |
| 5 | Without a uterus I would feel ashamed of my body |
| 6 | I feel the uterus is important for my relationship with my partner |
| 7 | A partner would find me unattractive after the removal of my uterus |
| 8 | I am worried that the removal of the uterus will make the sex live uncomfortable for me |
| 9 | I am worried that sexual intercourse could be uncomfortable for a partner after the removal of the uterus |
| 10 | Removing my uterus would make me sad as I would lose the ability to bear children |
| 11 | I wouldn´t tell it my friends and/or members of my family that my uterus has been removed |
| 12 | After the removal of my uterus I would feel older |
|  | “Benefit-of not having-uterus” scale |
| 1 | I assume the removal of the uterus would reduce the risk of pelvic cancers |
| 2 | I assume the removal of the uterus would reduce the risk of vaginal bleeding |
| 3 | I assume the removal of the uterus would help me to reduce chronic pelvic pains |
| 4 | I assume that without a uterus I would have less general health problems |
| 5 | I believe the uterus is useless to women who have completed childbearing |
| 6 | I believe removal of uterus would make prolapse treatments more effective |
| 7 | I think it is convenient no longer having to worry about birth control after removal of the uterus |
| 8 | I assume that my sex life will improve after removal of the uterus |
| 9 | I feel the prolapsed uterus is a sick organ in my body |
| 10 | I believe removal of the prolapsed uterus would make my body more attractive |
| 11 | I believe that I would have to see my gynaecologist less frequently after having my uterus removed |
| 12 | My family and/or friends have had a positive experience of removal of the uterus |
